# Mice Lacking Hbp1 Function Are Viable and Fertile

**DOI:** 10.1371/journal.pone.0170576

**Published:** 2017-01-20

**Authors:** Cassy M. Spiller, Dagmar Wilhelm, David A. Jans, Josephine Bowles, Peter Koopman

**Affiliations:** 1 Institute for Molecular Bioscience, The University of Queensland, Brisbane, Queensland, Australia; 2 School of Biomedical Sciences, Monash University, Clayton, Victoria, Australia; 3 School of Biomedical Sciences, The University of Queensland, Brisbane, Queensland, Australia; University Hospital of Münster, GERMANY

## Abstract

Fetal germ cell development is tightly regulated by the somatic cell environment, and is characterised by cell cycle states that differ between XY and XX gonads. In the testis, gonocytes enter G_1_/G_0_ arrest from 12.5 days *post coitum* (dpc) in mice and maintain cell cycle arrest until after birth. Failure to correctly maintain G_1_/G_0_ arrest can result in loss of germ cells or, conversely, germ cell tumours. High mobility group box containing transcription factor 1 (HBP1) is a transcription factor that was previously identified in fetal male germ cells at the time of embryonic cell cycle arrest. In somatic cells, HBP1 is classified as a tumour suppressor protein, known to regulate proliferation and senescence. We therefore investigated the possible role of HBP1 in the initiation and maintenance of fetal germ cell G_1_/G_0_ arrest using the mouse model. We identified two splice variants of *Hbp1*, both of which are expressed in XY and XX fetal gonads, but only one of which is localised to the nucleus in *in vitro* assays. To investigate *Hbp1* loss of function, we used embryonic stem (ES) cells carrying a Genetrap mutation for *Hbp1* to generate mice lacking Hbp1 function. We found that *Hbp1*-genetrap mouse mutant germ cells proliferated correctly throughout development, and adult males were viable and fertile. Multiple *Hbp1-LacZ* reporter mouse lines were generated, unexpectedly revealing *Hbp1* embryonic expression in hair follicles, eye and limbs. Lastly, in a model of defective germ cell G_1_/G_0_ arrest, the *Rb1*-knockout model, we found no evidence for *Hbp1* mis-regulation, suggesting that the reported RB1-HBP1 interaction is not critical in the germline, despite co-expression.

## Introduction

Germ cells are highly specialized cells that are uniquely capable of undergoing meiosis and represent our means to reproduce. During embryo development, two distinct cell cycle modes characterize the sex-specific pathways of germ cell differentiation. From 12.5 dpc in mice, germ cells enter G_1_/G_0_ arrest, signifying commitment to spermatogenesis [1], while entry into meiosis prophase I in the ovary signifies commitment to oogenesis [2]. The somatic cell environment of the gonads directs these two germ cell fates. Retinoic acid has been shown to modulate meiosis entry in the ovary, while being antagonistic to pro-spermatogonia development [3–5]. In the testis, very little is known regarding germ cell entry and maintenance of G_1_/G_0_ arrest. In humans, failure of this process to occur correctly has been linked to testicular germ cell tumours and their precursor, germ cell neoplasia *in situ* [6, 7]. This connection provides strong motivation for investigating cell cycle regulation in these specialised cells.

With no clear effector molecule or signalling pathway that seems likely to fulfil the role of inducing mitotic arrest in germ cells, investigations in many laboratories used microarray analysis and subtraction screens to identify genes expressed sex-specifically at the appropriate time. Using a subtraction screen, Smith and colleagues (2004) identified *Hbp1*, encoding the transcription factor high mobility group box containing transcription factor 1 (HBP1), as being expressed during the onset of mitotic arrest in mouse fetal testes [8]. From these data and what is currently known of HBP1 function in other systems, we hypothesised that HBP1 may be involved in the initiation or maintenance of mitotic arrest in fetal male germ cells.

HBP1 is a transcription factor that was first identified as an interacting factor of the cell cycle regulator retinoblastoma 1 (RB1) via a yeast two-hybrid screen [9]. HBP1 was subsequently shown to contain two RB1 interaction motifs, facilitating interaction with RB1 and related factor p130 [9, 10]. HBP1 shares DNA binding domain homology with members of the T cell-specific transcription factor lymphoid enhancer factor (TCF/LEF) [11] and the SRY-related HMG box (SOX) [12] families. The prominent feature of these families is their high mobility group (HMG) domain that sequence-specifically interacts exclusively with DNA [13]. Recent studies have identified a diverse range of gene targets for HBP1, including the MYC family (*Myc* and *Mycn*), cell cycle genes (*cyclin D1*), NADPH oxidase pathway (*p46 phox*), chromatin remodelling (*histone H1*^*0*^) and myeloperoxidase (reviewed in [14]). Interestingly, in humans, MYC and deregulation of the germ cell mitosis-to-meiosis switch have been implicated in the genesis of germ cell neoplasia *in situ* [15, 16]. Via protein-protein interactions with cell cycle regulators (RB1, p130) and repression of specific gene targets, the role of HBP1 is to set up proliferation barriers and trigger differentiation in various cell contexts. Indeed, over-expression of HBP1 *in vitro* and *in vivo* results in cell cycle arrest even in the presence of optimal proliferation signals [10, 14, 17].

In this study we focused on *Hbp1* expression and function in male fetal germ cells during the period of G_1_/G_0_ arrest (12.5 dpc—birth). We provide the first report of a splice variant of *Hbp1* that is expressed in male and female gonads, and describe the phenotype of a novel *Hbp1*-knockout mutant mouse line. We also generated and analysed two *Hbp1-LacZ* reporter lines to uncover further unreported embryonic tissue expression, and investigated a role for *Hbp1* in a model of defective germ cell G_1_/G_0_ arrest—the *Rb1*-knockout mouse.

## Materials and Methods

### Ethics statement

All mouse strains and experimental protocols were conducted in accordance with the Animal Ethics Committee of the University of Queensland (approval # IMB/131/09/ARC and SBMS/121/16/NHMRC/ARC/BREED).

### Animals

Animals were maintained in a recurrent photo cycle of 12h on-off in temperature controlled (22°C ± 2°C) rooms within the University of Queensland Biological Resource Centre. Mice received a diet of irradiated Meat Free Rat and Mouse Diet (Specialty Feeds, Glen Forrest, Australia) and fresh autoclaved water *ad libitum*. Mice were housed in filter-top static micro isolator cages with fine cord cob bedding and crinkle nest enrichment. Physical condition was monitored daily and provisions were in place to ensure any animals exhibiting adverse health received veterinary advice and/or immediate euthanasia to minimise pain and/or distress. Within the HGT colony, one mouse developed testicular teratoma and was euthanised by cervical dislocation immediately at external visualisation of the mass (detailed in Results). All other mice were euthanised by cervical dislocation at the experimental endpoint. Strains included: outbred CD1 mice and the *W*^*e*^*/W*^*e*^ mutant strain on outbred Swiss background (Quackenbush strain) [18]. *Rb1*^*-/-*^ mice were generated as previously described [19, 20] and maintained on a C57BL/6 background. Genotyping was performed as described previously [20].

### Generation of the 2KbHbp1P_pHSP68_LacZ mouse lines

The 2 kb *Hbp1* proximal promoter region was amplified from C57Bl/6 genomic DNA using the Expand High Fidelity PCR System (Roche, Indianapolis, USA) using primers *Hbp1P2kb-F* and *Hbp1P2kb-R* (S1 Table). The region included -2121 bp to +1 bp relative to the *Hbp1* transcription start site and was cloned into a modified pBluescript vector containing LacZ driven by the minimal HSP68 promoter. Transgenic mouse lines were produced by standard methods [21]. Tail tip biopsies were genotyped for the presence of the β-galactosidase cassette using primers *LacZ-F* and *LacZ-R* (S1 Table). Positive *Hbp1*^*lacZ*^ founders were then crossed to C57Bl/6 mice to generate lines, positive studs were identified and mated with C57Bl/6 wildtype females and embryos were collected for analysis.

### Generation of Hbp1-genetrap mice

The Bay Genomics ES Cell Line RRF373 containing the β-geo splicetrap vector, pGT0Lxf [22] was obtained from the Mutant Mouse Regional Resource Centers (MMRRC, Davis, USA) (www.mmrrc.org/). The E14 mutant ES cell line (G418-resistant; 129P2/Ola) was used for blastocyst injection into C57Bl/6 blastocysts. Germline transmission was achieved by mating chimeric males to wild-type C57Bl/6 females, heterozygotes detectable by agouti coat colour. Intercrossing of the F_1_ heterozygotes generated 129P2/Ola/C57Bl/6 mixed background offspring and the line continued to be backcrossed to C57BL/6 over at least 7 subsequent generations.

Mice were genotyped by PCR from genomic DNA extracted by tail tip biopsy. Two separate PCR protocols were employed that allowed for detection of the β-geo cassette alone (primers *LacZ-F* and *LacZ-R*) in addition to a product spanning *Hbp1* exon 5 and the β-geo cassette (primers *Ex5_Geo-F* and *Ex5_Geo-R*). Primers are listed in S1 Table.

### Embryo collection and organ culture

Embryos were collected from timed matings, with noon of the day on which the mating plug was observed designated 0.5 dpc. Embryo sex was determined by gonadal morphology (the presence or absence of testis cords). *Rb1*^*-/-*^ gonads were cultured as previously described [20, 23].

### Cell lines

Human embryonic kidney (HEK293) cells [24] were cultured at 37°C, 5% CO_2_ in Dulbecco’s Modified Eagle Medium including 10% fetal calf serum. HEK293 cells were grown to 90% confluency on cover slips in 6-well plates, transfected with 4 μg of each *Fl-Hbp1* or *ΔHbp1* expression vector using Lipofectamine 2000 reagent (Invitrogen, Carlsbad, USA) as per manufacturers’ instructions. Cells were analysed after 24 h incubation at 37°C.

### Whole mount in situ hybridisation

A 903 bp Fl-Hbp1 and a 573 bp ΔHbp1 3’UTR fragment were cloned from 12.5 dpc testis. Primers were *Fl-Hbp1-F* and *Fl-Hbp1-R* and *ΔHbp1-F* and *ΔHbp1-R*, listed in S1 Table. The amplified fragments were cloned into the pGEM-T-Easy vector (Promega, Madison, USA) and verified by sequencing. The sense and anti-sense probes were synthesised using T7 and SP6 RNA polymerases through *in vitro* transcription. Gonads/mesonephroi were dissected and fixed in 4% paraformaldehyde (PFA) in phosphate-buffered saline (PBS) for several hours at 4°C. Whole mount *in situ* hybridization (ISH) with digoxygenin (DIG) labelled RNA probes was carried out as described by [25]. The RNA probe was detected by incubation with BM Purple, AP Substrate (Roche, Indianapolis, USA).

### Quantitative real time PCR and statistical analysis

Embryonic gonads (without mesonephros) were dissected in PBS and total RNA was immediately isolated using the Micro RNA kit (Qiagen, Hilden, Germany) as per manufacturers’ instructions including the optional *DNaseI* genomic DNA degradation step. cDNA was synthesised from 1 μg of RNA by reverse transcription (Superscript III, Invitrogen, Carlsbad, USA) using random primers (Promega, Madison, USA) according to manufacturers’ instructions. The ABIPrism-7000 Sequence Detector System was used to analyse relative cDNA levels. Primers are listed in S1 Table. All quantitative RT-PCR experiments were performed in triplicate and repeated on three separate biological samples. Results are represented as mean ±S.E.M of the experiments. Briefly, samples were analysed in 25 μl reactions containing 1 μl cDNA prepared as described above, SYBR Green PCR Master Mix (Applied Biosystems, Foster City, USA) and 3.75 μM each of forward and reverse primers. Cycling conditions began with an initial 10-min step at 95°C followed by 40 cycles of 95°C for 15 sec and 60°C for 1 min in a two-step thermal cycle. Dissociation curves were analysed for each primer set and cDNA samples were normalised against both *18S rRNA* and *Rps29* using the 2^-ΔΔCT^ method. Statistical analysis was performed using Prism. Pairwise comparisons were analysed using two-tailed student t-tests.

### HBP1-MYC constructs

Truncated *Hbp1* cDNA constructs were amplified from 12.5 dpc testis cDNA using the Expand High Fidelity PCR System (Roche, Indianapolis, USA) with the common forward primer All-*Hbp1-MYC-F* containing a *BamHI* restriction site and individual reverse primers containing an *XhoI* restriction site, *FlHbp1-MYC-R* and *ΔHbp1-MYC-R* (S1 Table). These fragments were cloned into a modified pcDNA3.0 vector generating an N-terminal MYC-tagged protein and sequenced for verification.

### Whole mount LacZ staining

Whole embryos and embryonic tissues were collected in ice-cold PBS and fixed for several hours in glutaraldehyde fixative (1% formaldehyde, 0.2, glutaraldehyde, 2 mM MgCl_2_, 5 mM EGTA and 0.02% NP-40 in PBS) at room temperature. Whole mount LacZ staining of these tissues was then performed as follows: Embryos were washed three times in PBS and stained for ß-galactosidase in 5mM K_3_Fe(CN)_6_, 5mM K_4_Fe(CN)_6_, 2mM MgCl_2_, 0.02% NaDeoxycholate, 0.02% NP-40 and 1mg/ml X-gal in phosphate buffered saline overnight at 37°C. After desired colour development was achieved, embryos were washed three times in PBS and post-fixed in 4% PFA in PBS for 30 min at 4°C.

### Histological and immunofluorescence analysis

Embryos and gonads were dissected in ice-cold PBS and fixed in 4% PFA in PBS for several hours. Samples were then embedded in paraffin before sectioning at 7 μm on a microtome (Leica, Wetzlar, Germany). For morphological analysis sections were deparaffinised and stained with haematoxylin and eosin (H&E). Immunofluorescence with transfected cells [26] and on sections of mouse embryos [27] was performed as described previously.

Primary antibodies and dilutions used were: Anti-MYC (Cell Signalling Technology, Danvers, USA) 1:1000, anti-AMH (Santa Cruz Biotechnology, Dallas, USA) 1:200, anti-3βHSD (Prof J I Mason, The University of Edinburgh) 1:100, anti-MVH (AbCam, Cambridge, UK) 1:200, anti-E-cadherin (BD Bioscience, San Jose, USA) 1:200, anti-Ki67 (Clone TEC-3, DakoCyomation, Glostrup, Denmark) 1:50, DAPI (Sigma Aldrich, St Louis, USA) 1:2000. Secondary antibodies and dilutions were all from Molecular Probes (Eugene, USA) and used at 1:200: goat anti-rabbit Alexa Flour 488, donkey anti-rat Alexa Fluor 594, goat anti-mouse Alexa Fluor 594 and donkey anti-goat Alexa Fluor 488.

H&E section images were captured on an Olympus (Shinjuku, Japan) BX51 BF/DF microscope. Whole mount LacZ imaging was performed on an Olympus (Shinjuku, Japan) SZX-12 stereo dissecting microscope. Photographs were taken with an Olympus (Shinjuku, Japan) DP-70 camera using the default settings of the Olympus software DP Controller (version 2.1.1.183). Immunofluorescence-labelled sections were imaged using Zeiss (Oberkochen, Germany) LSM-510 confocal microscope.

### Western blotting

Standard Western blot analysis was performed with the following antibodies and dilutions: Anti-HBP1 (N-20, Santa Cruz Biotechnology, Dallas, USA) 1:200, anti-alpha-tubulin (Clone B-5-1-2, Sigma Aldrich, St Louis, USA) 1:500, donkey anti-goat conjugated to horse radish peroxidase (HRP) (Jackson Laboratories, Bar Harbor, USA) 1:2000, goat anti-mouse-HRP (Sigma Aldrich, St Louis, USA) 1:2000.

## Results

### Hbp1 is expressed during mouse gonad development

**Fig 1:**
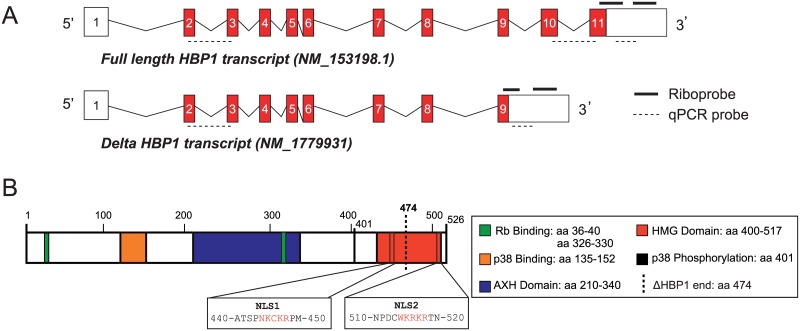
Alternative splicing of *Hbp1* transcripts. **(A)** Gene structure of the two *Hbp1* splice variants. The full-length transcript comprises 11 exons and the truncated transcript is composed of the first 9 exons. Both transcripts contain identical 5’UTRs in addition to distinct 3’UTRs (open boxes), the specific riboprobes and real time PCR probes are depicted. **(B)** Location of HBP1 protein domains and nuclear localisation signals (NLS).

Ensembl genome browser database analysis of the *Hbp1* sequence revealed two putative *Hbp1* splice variants. The shorter transcript (referred to as delta Hbp1; *ΔHbp1*) omits exons 10 and 11 of the full-length transcript (referred to as full length Hbp1; *FL-Hbp1*), resulting in and a truncated coding region and an alternative 3’ untranslated region (Fig 1A). The encoded proteins contain several conserved domains including RB binding, p38 docking and phosphorylation sites in addition to the HMG domain, which is truncated at amino acid (aa) 474 in the smaller protein (Fig 1B).

**Fig 2:**
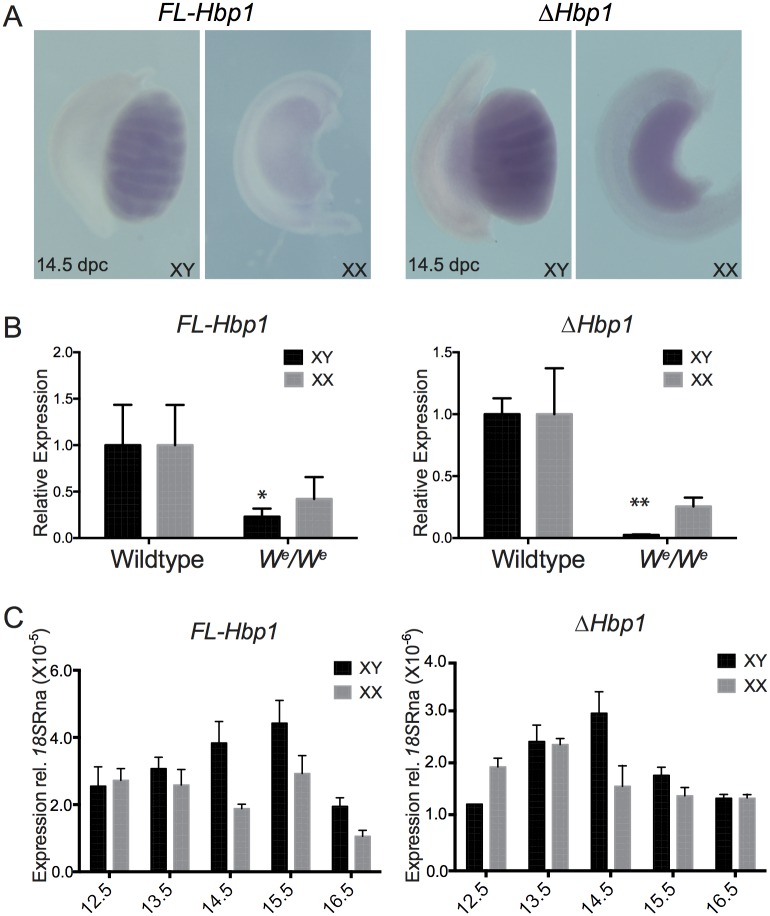
Detection of *Fl-Hbp1* and *ΔHbp1* transcripts in wildtype and *W*^*e*^*/W*^*e*^ gonads. **(A)** Detection of *Fl-Hbp1* and *ΔHbp1* in 14.5 dpc XX and XY gonads using whole mount in situ hybridisation **(B)** qRT-PCR analysis detected lower levels of *Fl-Hbp1* and *ΔHbp1* gene expression in 13.5 dpc XY and XX *W*^*e*^*/W*^*e*^ mutant gonads which lack germ cells. Expression was normalised to *18S* RNA (mean ± S.E.M of three independent experiments, each performed in triplicate) and wildtype controls set to 1. *P < 0.05; **P < 0.005. **(C)** qRT-PCR detected the *Fl*-*Hbp1* and *ΔHbp1* transcripts in gonad-only cDNA samples from 12.5–16.5 dpc in XX and XY gonads. Expression was normalised to *18S* RNA (mean ± S.E.M of three independent experiments, each performed in triplicate).

We investigated the expression of both transcripts in developing mouse gonads at 14.5 dpc using whole mount ISH. Using antisense riboprobes hybridizing to the unique 3’UTRs, we detected expression of *FL-Hbp1* in the testis only, whereas *ΔHbp1* was expressed in both XY and XX gonads (Fig 2A). Sense control riboprobes did not yield any staining (S1 Fig). To determine if the expression was present in germ and/or somatic cells, we analysed expression levels of both *Hbp1* transcripts in the *W*^*e*^*/W*^*e*^ mutant mouse model using quantitative real time RT-PCR (qRT-PCR). Mice homozygous for mutations in the *W* (dominant white spotting) locus (*W*^*e*^*/W*^*e*^) lack germ cells and die *in utero* at 14.5 dpc due to macrocytic anaemia [18]. Analysis of *FL-Hbp1* and *ΔHbp1* expression in 13.5 dpc *W*^*e*^*/W*^*e*^ mutant gonads revealed that expression was significantly decreased in XY samples lacking germ cells indicating that it is largely restricted to, or dependent on, germ cells in XY gonads (Fig 2B). In XX gonads, expression was not significantly decreased, suggesting there may be a larger somatic component of *FL-Hbp1* and *ΔHbp1* expression in this organ (Fig 2B). Next, we used qRT-PCR to compare levels of *Hbp1* expression in wild-type testes and ovaries over a wider time-course. In the XY gonad, the highest expression of *FL-Hbp1* and *ΔHbp1* was detected at 15.5 dpc and 14.5 dpc respectively. In the ovary, expression of *FL-Hbp1* and *ΔHbp1* appeared to generally decline over the timecourse, with lowest expression detected at 16.5 dpc (Fig 2C). Attempts to localise HBP1 protein expression by immunofluorescence using an antibody were unsuccessful, so we turned to *in vitro* experiments.

### HBP1 variants display different cellular localization

**Fig 3:**
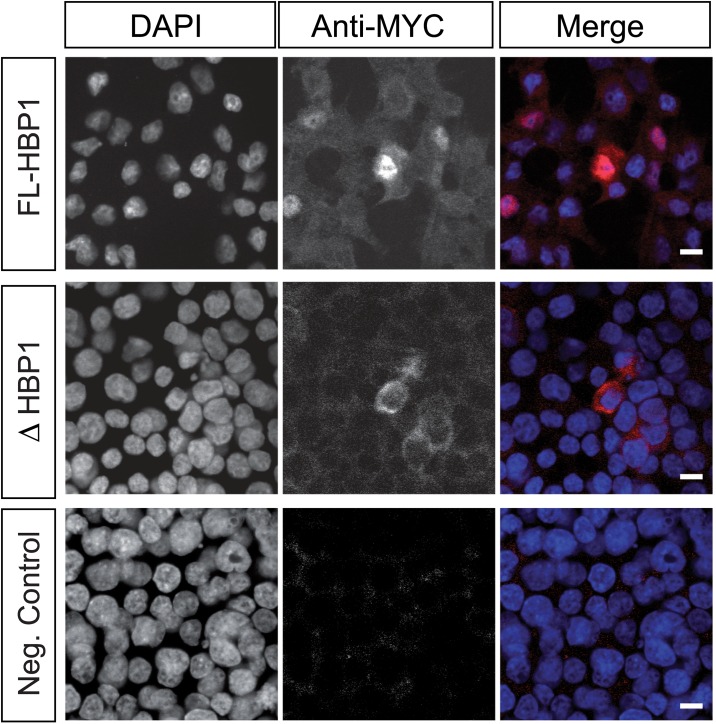
Cellular localisation of Fl-HBP1 and ΔHBP1. Myc-tagged HBP1 variants were transfected into HEK293 cells and immunostained for MYC expression. Cells were counterstained with DAPI to identify the nuclei and were visualised using confocal microscopy. FL-HBP1 was localised to the nucleus with some cytoplasmic staining also detected. ΔHBP1 remained in the cytoplasm. The control represents cells transfected without DNA. Scale Bar = 10μm.

As HBP1 is a transcription factor, it must gain access to the nucleus to perform its function. We therefore investigated the cellular localisation of both variants in cell culture. Bioinformatic analysis of the HBP1 amino acid sequence revealed the presence of two un-reported nuclear localisation signals (NLSs) flanking the HMG domain at positions aa 444–448 (NKCKR) and aa 514–518 (WKRKR) (Fig 1B). These putative NLSs are conserved within families of HMG-containing proteins including the SOX family of transcription factors [28]. As *ΔHbp1* lacks the second NLS, we hypothesised that *ΔHbp1* would not be efficiently localised to the nucleus. To investigate this, we generated both variants in-frame with a MYC tag and transfected them into HEK293 cells. Analysis by MYC immunofluorescence revealed mainly nuclear and some cytoplasmic localization of FL-HBP1 (containing both NLS sequences), but exclusively cytoplasmic localisation of ΔHBP1 (Fig 3). These data suggest that the C-terminal NLS is necessary for HBP1 nuclear localisation and importantly, has revealed a significant functional difference between FL-HBP1 and ΔHBP1.

### The Hbp1 promoter is active in vivo

Next, we employed a transgenic approach using *in vivo* ß-galactosidase expression driven by the *Hbp1* promoter for two reasons. First, this strategy allows identification of regulatory regions required for germ cell expression of *Hbp1 in vivo*, and secondly, the generation of a germ cell-LacZ expressing transgenic line that can be used in gonadal explant culture as an indication of G_1_/G_0_ arrested germ cells would provide an excellent tool for germ cell research. 2 kb of the *Hbp1* proximal promoter was amplified and cloned into the pHSP68_LacZ expression vector [29]. Following pro-nuclear injection, two founding *Hbp1*^*Lacz*^ lines were established and characterised by ß-galactosidase staining.

**Fig 4:**
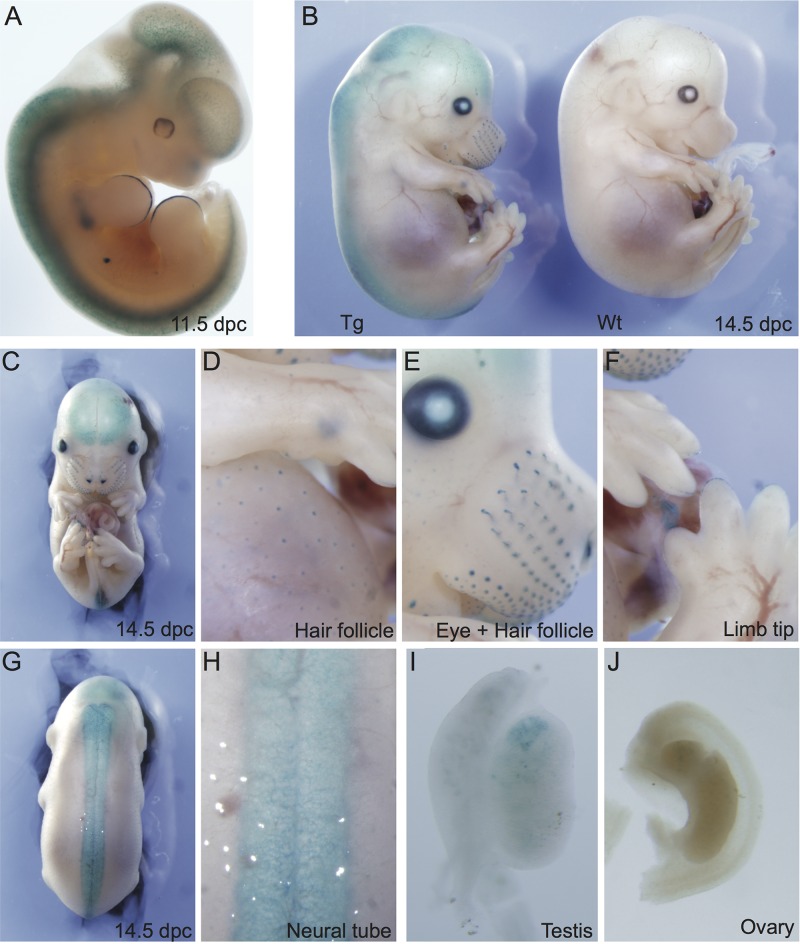
LacZ expression analysis of *Hbp1*^*lacZ_1*^. LacZ expression, driven by the 2 kb *Hbp1* proximal promoter, was assessed at embryonic stages 11.5 **(A)** and 14.5 dpc **(B)**; wildtype (Wt); transgenic (Tg). Promoter activity was also detected in 14.5 dpc somatic tissues including the forebrain **(C),** hair follicles **(D)**, eye **(E)**, limb tips **(F)** and neural tube **(G,H)**. Expression was also detected at 14.5 dpc in the XY **(I)**, but not XX **(J)** gonad.

The first *Hbp1*^*lacZ*^ founder line (*Hbp1*^*lacZ_1*^) displayed specific staining in several fetal somatic tissues including the brain, neural tube, eye, limb tips and hair follicles (Fig 4). Importantly, expression of LacZ driven by the 2 kb promoter region of *Hbp1* was also detected in 14.5 dpc XY gonads but not in ovaries at this stage. The XY gonadal staining was not strong enough to identify individual LacZ-expressing cell types following sectioning (S2 Fig).

Analysis of the second *Hbp1*^*lacZ*^ founder (*Hbp1*^*lacZ_2*^) revealed comparable LacZ expression to founder 1 in several fetal somatic tissues including the hair follicles and eye in addition to the forebrain (S3 Fig), suggesting that this might be a true reflection of the activity of this *Hbp1* promoter region *in vivo*. Differences were seen in the cell types expressing LacZ within the neural tube, as *Hbp1*^*lacZ_2*^ stained within the dorsal root ganglia rather than the epithelia as seen for *Hbp1*^*lacZ_1*^. In addition to the tip of each expanding digit, expression was also detected in the limb proper, and there was an absence of LacZ expression in the hindbrain in *Hbp1*^*lacZ_2*^. Unlike *Hbp1*^*lacZ_1*^, there was no detectable LacZ expression within the fetal XY and XX gonads (S3 Fig).

### Analysis of Hbp1-mutant mice

**Fig 5:**
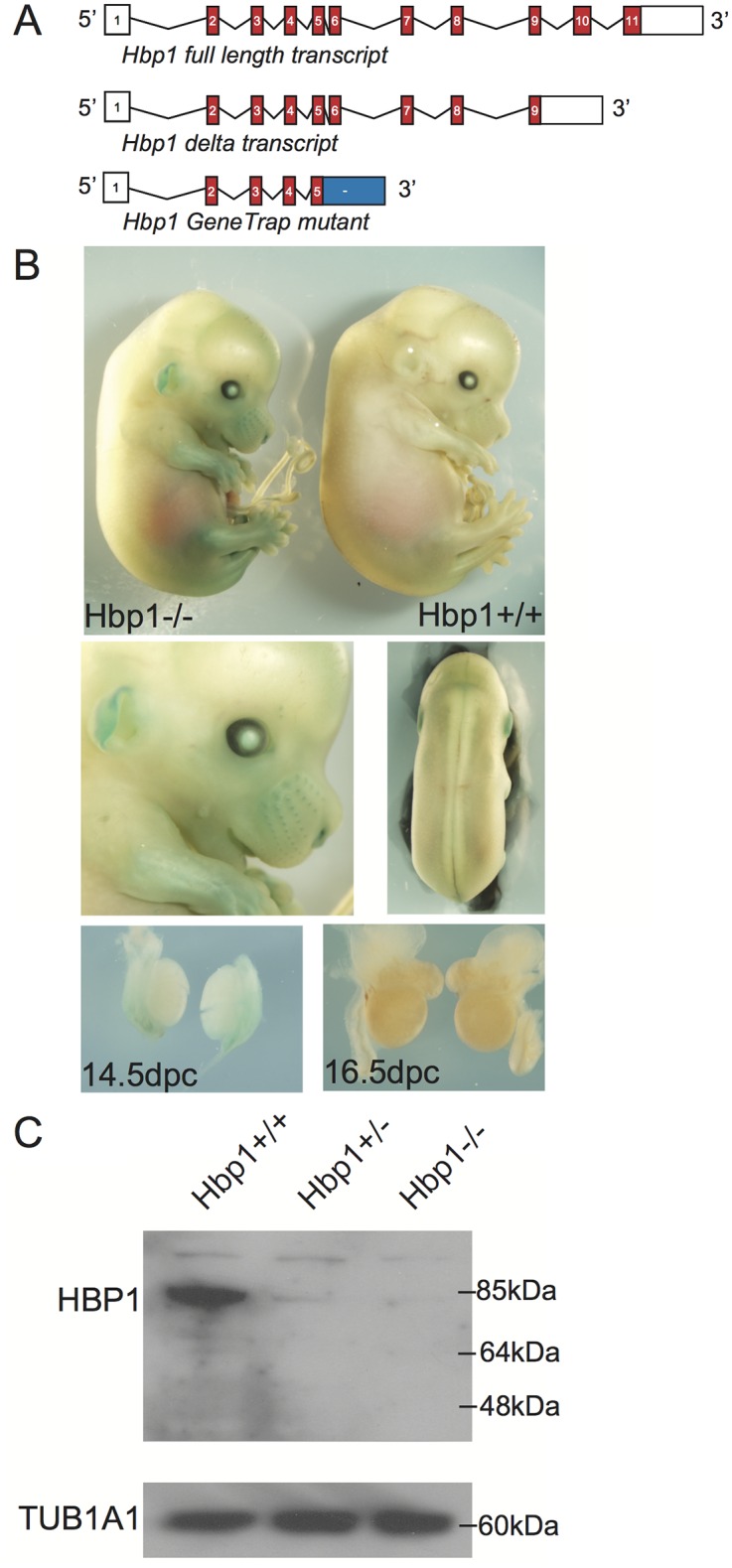
Morphological and protein expression analysis of *Hbp1*^*+/+*^ and *Hbp1*^*-/-*^ mutant embryos. **(A)** Gene structure of the two *Hbp1* splice variants and the *Hbp1* gene-trap variant which contains a ß-galactosidase cassette inserted directly downstream of exon 5. **(B)** Whole 14.5 dpc *Hbp1*^*+/+*^ and *Hbp1*^*-/-*^ embryos were stained for LacZ expression. LacZ was detected in *Hbp1*^*-/-*^ embryos in the eye, hair follicles, limbs, ears and mesonephros, but not testes at 14.5 or 16.5 dpc. **(C)** Western blot analysis was performed on whole 14.5 dpc *Hbp1*^*+/+*^, *Hbp1*^*+/-*^ and *Hbp1*^*-/-*^ embryo lysates. Anti-HBP1 detected reduced levels of HBP1 protein in the *Hbp1*^*+/-*^ and *Hbp1*^*-/-*^ samples compared to *Hbp1*^*+/+*^ control. TUBA1A was used as the loading control.

We next generated *Hbp1* loss-of-function mice in order to investigate the effects on embryonic development. *Hbp1* “gene-trap” mutant ES cells, containing a ß-galactosidase cassette insertion directly downstream of exon 5, were obtained from Bay Genomics (Fig 5A). The nature of the *Hbp1* genetrap mutation potentially gives rise to a truncated protein containing the first 5 exons (218 aa). An expression construct of the *Hbp1*-mutant variant was cloned and expressed in HEK293 cells with a N-terminal MYC tag and revealed that the shortened protein is expressed exclusively within the cytoplasm (S4 Fig), suggesting that if this product is translated *in vivo*, it cannot have a nuclear function, including binding to DNA.

Following blastocyst injection of the *Hbp1*-mutant ES cells, breeding of chimeric male offspring resulted in germline transmission of the *Hbp1*-genetrapped allele. Heterozygotes for the mutation (*Hbp1*^*+/-*^) were viable and fertile and subsequent *Hbp1*^*+/-*^ x *Hbp1*^*+/-*^ matings yielded *Hbp1*^*-/-*^ embryos for analysis. As with the adult heterozygous mutants, the homozygous embryos displayed normal gross morphology of all somatic tissues (Fig 5B). Both heterozygous and homozygous males were fertile fathering an average of 6.7 ±2.8 (*n* = 7) and 7.3 ±1.2 (*n* = 3) pups per litter respectively. Accordingly, adult testis gross morphology was comparable between wildtype controls and homozygous mutants with testis/body weight ratios of 0.24 ±0.03 and 0.28 ±0.01 respectively (*n* = 3). We observed one instance of testicular teratoma in a chimeric male founder (S5 Fig), but this phenomenon did not affect any of the *Hbp1*^*+/-*^ males in the established colony (0/53 males).

We also stained *Hbp1*^*+/-*^ embryos for LacZ expression taking advantage of the ß-galactosidase cassette insertion. Similarly to the *Hbp1* promoter transgenic lines (*Hbp1*^*lacZ_1*^ and *Hbp1*^*lacZ-2*^), LacZ expression was detected in hair follicles, eye and limbs of embryos at 14.5 dpc. In contrast, no ß-galactosidase activity was detected in fetal gonads at 14.5 and 16.5 dpc, or in the neural tube or brain, unlike the promoter transgenic lines (Figs 5B and 4, S3 Fig). Although these analyses suggested that *Hbp1* is endogenously expressed during the development of hair follicles, eye and limbs, adult *Hbp1*^*+/-*^ (*n* = 67) and *Hbp1*^*-/-*^ (*n* = 12) mice had no reported defects with hair growth, eyesight or limb morphology.

In order to determine if HBP1 protein was translated in the heterozygous and homozygous mutants, whole 14.5 dpc embryo lysates were investigated by Western blot analysis using an HBP1 polyclonal antibody that recognises an epitope near the N-terminus of the protein. This analysis revealed greatly reduced levels of expression in both the *Hbp1*^*+/-*^ and *Hbp1*^*-/-*^ embryos compared to wildtype *Hbp1*^*+/+*^ control (Fig 5C). No lower molecular weight protein was apparent in the *Hbp1*^*+/-*^ and *Hbp1*^*-/-*^ embryos, suggesting that the truncated 218aa protein (detectable with the polyclonal antibody used; predicted molecular weight of 53 kDa) is either not translated, or is readily degraded.

We next made a thorough assessment of testis development in *Hbp1*^*-/-*^ mutant embryos. Because the *Hbp1* gene-trap mutation was ubiquitous in these embryos, somatic cells were also assessed for any phenotype that may arise due to the ablation of HBP1. Using H&E staining we found normal testis morphology at 12.5, 14.5 and 16.5 dpc (S6 Fig). Using immunofluorescence, we further found that expression of somatic cell markers AMH (Sertoli cells) and 3ßHSD (Leydig cells) were similar between genotypes at 14.5 dpc (S7 Fig).

**Fig 6:**
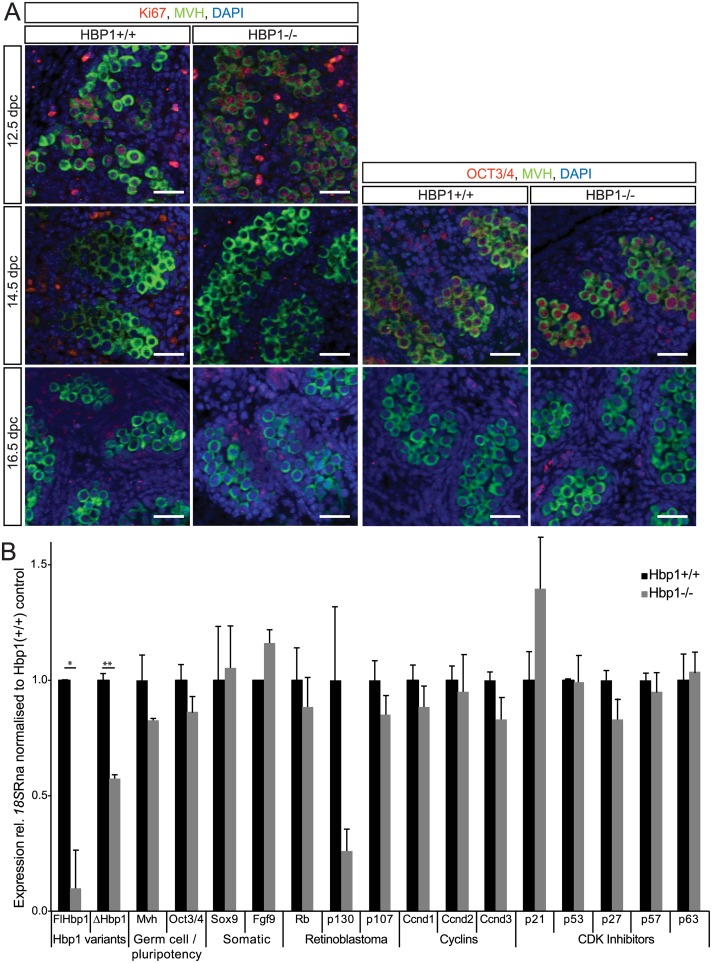
Immunohistochemical and gene expression analysis of *Hbp1*^*+/+*^ and *Hbp1*^*-/-*^ embryonic gonads. **(A)** Proliferation marker Ki67 in germ cells (MVH-positive cells) was detected in both *Hbp1*^*+/+*^ and *Hbp1*^*-/-*^ gonads at 12.5 dpc, but was absent in germ cells of 14.5 and 16.5 dpc gonads of both genotypes. Pluripotency marker OCT3/4 was expressed similarly in germ cells (MVH-positive cells) in *Hbp1*^*+/+*^ and *Hbp1*^*-/-*^ gonads at 14.5 dpc and was undetectable at 16.5 dpc. Scale bar = 50μm. **(B)** qRT-PCR analysis of *Hbp1*^*+/+*^ and *Hbp1*^*-/-*^ 16.5 dpc gonad samples revealed comparable expression between of various germ cell and somatic cell markers. Germ cell markers: *Mvh*, *Oct3/4*and G_1_/G_0_ arrest indicator *p63*, somatic cell markers *Fgf9* and *Sox9*, Retinoblastoma family members *Rb1*, *p130* and *p107*, and cell cycle regulators *p21*, *Ccnd1-3* and *p53* displayed no significant difference between *Hbp1*^*+/-*^ *and Hbp1*^*-/-*^ samples. Samples normalised to *18S* RNA (mean ± S.E.M of three independent experiments, each performed in triplicate). *P < 0.05; **P < 0.005; ns = not significant.

To investigate the hypothesis that HBP1 controls G_1_/G_0_ arrest in fetal XY germ cells, proliferation marker Ki67 was assessed in the germ cell population, marked by expression of MVH. Here we found that germ cells were proliferating (Ki67-positive) equally at 12.5 dpc in both *Hbp1*^*+/+*^ and *Hbp1*^*-/-*^ samples, but were not proliferating (Ki67-negative) at 14.5 and 16.5 dpc in either mutants or wild type controls (Fig 6A). Lastly, we assessed pluripotency marker OCT3/4 in the germ cell population. We detected OCT3/4 expression in germ cells of both genotypes at 14.5 dpc, but not at 16.5 dpc (Fig 6A). Although *Hbp1*^*-/-*^ XY gonads appeared to have a normal morphology and profile of marker protein expression as detected by immunofluorescence, we next investigated gene expression in these samples, in an effort to identify a subtler phenotype in the absence of *Hbp1*. We performed qRT-PCR for a variety of germ cell and cell cycle markers in 16.5 dpc gonad samples from which mesonephroi had been removed (Fig 6B). Consistent with the immunofluorescence data, there were no significant changes to the germ cell markers *Mvh* or *Oct3*/*4*, and *p63*, a marker of G_1_/G_0_ arrest, was also unchanged. Additionally, somatic markers *Fgf9* and *Sox9*, retinoblastoma family members *Rb1*, *p130* and *p107* and cell cycle regulators *p21*, *p27* and *p57* were comparable between controls and *Hbp1*^*-/-*^ samples. Therefore, having determined that the *Hbp1*-mutant embryos showed normal morphology, fertility and viability, we conclude that HBP1 is dispensable for correct embryo development and subsequent adult survival.

**Fig 7:**
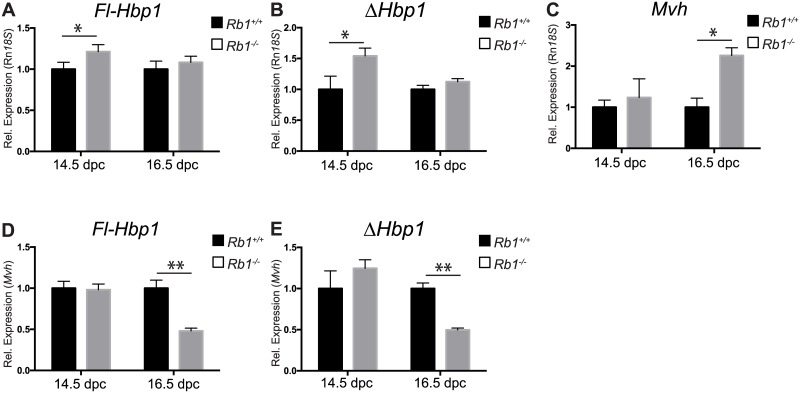
*Hbp1* expression in *Rb*^*+/+*^ and *Rb*^*-/-*^ mutant XY gonads. Using qRT-PCR analysis, normalizing gene expression to *18S*RNA, both *Fl-Hbp1* **(A)** and *ΔHbp1* **(B)** gene expression was significantly increased in *Rb*^*-/-*^ mutant XY gonads at 14.5 dpc. In 16.5 dpc *Rb*^*-/-*^ cultured XY gonads, there was no significant difference in either *Fl-Hbp1* or *ΔHbp1* gene expression, although germ cell marker *Mvh* was significantly increased at this timepoint (**C**). When gene expression was normalized to germ cell marker *Mvh*, both *Fl-Hbp1* **(D)** and *ΔHbp1* **(E)** gene expression was significantly decreased in 16.5 dpc *Rb*^*-/-*^ cultured XY gonads. (mean ± S.E.M of three independent experiments, each performed in triplicate; wildtype controls (*Rb*^*+/+*^) set to 1). *P < 0.05; **P < 0.005.

It remained possible that *Hbp1* might play a role in ameliorating the phenotypes of other experimentally-produced mouse mutants showing defective germ cell cycle control. In *Rb1* loss-of-function (*Rb1*^*-/-*^) mutant mice, some XY germ cells fail to enter G_1_/G_0_ arrest at 14.5 dpc unlike the wildtype control [20]. Despite this aberration, arrest is achieved in the total germ cell population by 16.5 dpc, likely due to compensation by CDK inhibitors *Cdkn1b* and *Cdkn2b* which are upregulated in mutant germ cells [20]. We investigated whether *Hbp1* might similarly be upregulated in the absence of *Rb1* in order to achieve eventual G_1_/G_0_ arrest in these cells. Using qRT-PCR, we found a small but significant increase in levels of both *FL-Hbp1* and *ΔHbp1* in *Rb1*^*-/-*^ gonads at 14.5 dpc (Fig 7A and 7B). However, because germ cell number is increased in *Rb1*^*-/-*^ mutants due to their cell cycle defect (measured by *Mvh* expression; Fig 7C), we also normalized *FL-Hbp1* and *ΔHbp1* expression to *Mvh* to account for this germ cell number increase. We found that *FL-Hbp1* and *ΔHbp1* expression was unchanged at 14.5 dpc but significantly decreased at 16.5 dpc in germ cells of *Rb1*^*-/-*^ mutant gonads (Fig 7D and 7E). These data suggest that in XY germ cells *Hbp1*, at least at the RNA level, is unlikely to contribute to the back-up mechanism performed by other CDK inhibitors to compensate for *Rb1* failure to achieve G_1_/G_0_ arrest by 16.5 dpc.

## Discussion

The embryonic differentiation of XX and XY germ cells diverges at 12.5 dpc with XX germ cell entry into the first phase of meiosis and XY germ cell entry into G_1_/G_0_ arrest [1]. XY germ cells are particularly fascinating as they maintain a prolonged period of G_1_/G_0_ arrest throughout development and postnatal life, prior to entering mitosis and meiosis after puberty [30]. Aberrations in the entry or maintenance of G_1_/G_0_ arrest have led to instances of both germ cell loss and conversely, proliferation and therefore cancer, such as observed in *Pten-/-* [31], *Dazl-/-* [32] and *Ter* mutations [33]. It is also believed that loss of correct germ cell-cell cycle control and differentiation is responsible for germ cell neoplasia *in situ* (previously known as carcinoma *in situ* [34]), the precursor cell to human testicular germ cell tumours, for which there is no suitable mouse model. Comparison of germ cell and somatic cell tumours has revealed both common and unique gene and protein expression patterns, including for cell cycle regulators such as RB1 [6, 7]. It is possible that germ cells are subject to a different type of cell cycle regulation, which would not be surprising considering their unique ability to undergo meiosis.

Although we do not know what factor(s) may be responsible for initiating this cell cycle state, several cell cycle factors have been identified as being up-regulated or activated during fetal germ cell G_1_/G_0_ arrest. Particularly, activation of p27(KIP1), p15(INK4b) and p16(INK4a) and RB1 occurs at this time, in addition to upregulation of *p21*(*Cdkn1a*) [20, 35]. While these kinases and phospho-proteins comprise the machinery used to achieve and maintain G_1_/G_0_ arrest, the regulation of these factors within germ cells remains unclear. The transcription factor HBP1, identified by our group in a subtraction screen [8], is an interesting candidate for germ cell-specific regulation of this event due to its established role in cell cycle arrest and wide range of interacting factors. The work described here characterised the expression and functional role of *Hbp1* in the initiation and maintenance of XY germ cell mitotic arrest.

Using several techniques, *Hbp1* expression was enriched within the XY gonad during G_1_/G_0_ arrest as reported previously [8]. Further to the analysis presented by Smith and colleagues (2004), qRT-PCR analysis revealed maximal expression of *FL-Hbp1* at 14.5–15.5 dpc in the XY gonads. As germ cells are reported to begin entry into G_1_/G_0_ arrest as early as 12.5 dpc [35, 36] it seems more likely that the role of HBP1 in these cells is related to the maintenance/reinforcement of this arrest rather than its initiation.

Expression of a second *Hbp1* splice variant was also detected in germ cells at this stage and we considered that it might reflect a novel mechanism of *Hbp1* regulation and function. Subcellular visualization *in vitro* revealed that only FL-HBP1 is translocated into the nucleus, likely requiring both NLSs within the HMG domain. The SOX family member SRY utilises two similar NLS sequences flanking its HMG domain which associate with importin beta for nuclear localisation and RanGTP for disassociation [28]; we predict a similar mechanism may be used by HBP1, however this is yet to be confirmed. In contrast to FL-HBP1, ΔHBP1, which lacks this C-terminal NLS, remained within the cytoplasm. This suggests that FL-HBP1 can function as a transcription factor in XY germ cells, while ΔHBP1 presumably has an alternate role, independent of DNA binding. Within the cytoplasm ΔHBP1 could potentially regulate levels of HBP1 binding partners such as RB1 and p130, or perform a dominant-negative function by preventing FL-HBP1 nuclear import, although no evidence for HBP1 dimerization has emerged to date. Identification of ΔHBP1 binding partner(s) may be informative in identifying a role for this factor.

Using transgenic reporter lines we investigated activity of the endogenous 2kb *Hbp1* proximal promoter. Because of potential integration effects from the reporter transgenic lines, we compared LacZ expression between these and with the genetrap *Hbp1* knockout line to uncover likely sites of endogenous *Hbp1* reporter activity during embryonic development. Comparison of all lines suggests potential action of HBP1 within the eye, hair follicles and limbs, which have not been reported to date. Several other tissues such as the forebrain and neural tube displayed reporter line-specific LacZ expression suggesting that the transgene was vulnerable to integration effects and/or that the entire *Hbp1* promoter contains repressive elements for these tissues outside of the 2kb promoter investigated.

Given the role of HBP1 in cell cycle arrest (reviewed in [12]), its XY germ cell expression, and *in vivo* transcriptional regulation determined using the LacZ reporter and genetrap lines; we predicted that *Hbp1* loss-of-function would display a fetal germ cell proliferation defect and possible aberrations in hair follicle, eye and limb development. The *Hbp1*-mutant mouse line we generated successfully ablated HBP1 protein and transcript levels, yet showed normal gross embryonic development and fetal germ cell G_1_/G_0_ arrest and differentiation. Specifically, germ cell identity, cell number and cell cycle state assessed by both mRNA and protein expression was comparable between mutants and controls. Adult *Hbp1*^*+/+*^ and *Hbp1*^*-/-*^ mutants were viable and fertile (at least until 6 months of age), with no gross defects in any tissue or organ observed, despite reports of *Hbp1* functioning in an array of adult tissues including muscle, adipocyte, erythroid and liver (reviewed in [12]).

Recently, Dong and colleagues (2016) reported a conditional *Hbp1* loss-of-function model. Following ubiquitous deletion using the *Ella*-Cre recombinase [37], they observed defects in the oocyte reserve in adult females [35]. This phenotype was attributed to altered mitochondrial function in granulosa cells, and confirmed by using conditional deletion in the granulosa cells and germ cells separately [38]. In the absence of *Hbp1*, for unknown reasons and despite a general increase in ovarian reserve due to decreased apoptosis, mutant females were infertile by 7 months. We might expect the same phenotype in the *Hbp1* genetrap-derived mutant mice analysed here, but although we routinely mated heterozygous and homozygous genetrapped *Hbp1* females at ages younger than 7 months, we did not analyse any mice beyond that age.

An *in vitro* interaction between HBP1 and RB1 has been reported multiple times in the literature. In muscle cells the specific ratio of RB1/HBP1 has been shown to affect the regulation of the MyoD family and differentiation in C2C12 cells [10, 12]. We previously reported the function of RB1 in affecting correct timing of G_1_/G_0_ arrest in XY fetal germ cells, as in its absence germ cells exhibit a delay in G_1_/G_0_ entry and subsequent increase in total numbers [20]. In this study we investigated a role for HBP1 in the *Rb1*^*-/-*^ model but found no evidence that transcriptional upregulation of *Hbp1* compensates for *Rb1* loss in the germ cells. Because *Hbp1*^*-/-*^ germ cells affect correct G_1_/G_0_ arrest, it seems that, *in vivo*, the germline does not utilize an RB1-HBP1 interaction, or it has little biological significance, despite co-expression of both factors. Further analysis of HBP1 and RB1 protein level as well as binding partners in these mutants is required to confirm this conclusion.

In summary, we have profiled expression of *Hbp1* within the XY germ cell population at a time appropriate to the maintenance of their G_1_/G_0_ arrest. We have shown that FL-HBP1 can be translocated to the nucleus to presumably function as a transcription factor. *In vivo* expression of *Hbp1* was detected in the gonad as well as various other somatic tissues, suggesting that HBP1 may play a wider role in somatic tissue development/specification also. Genetic deletion of *Hbp1* revealed normal fetal germ cell cycle control, suggesting that its role, if any, in the germline is not fundamental.

## Supporting Information

S1 Fig Sense controls of *Fl-Hbp1* and *ΔHbp1* in-situ hybridisation probes. No signal was detected for either *Fl-Hbp1* or *ΔHbp1* sense control probes in 14.5 dpc gonads using whole mount in situ hybridisation.
(EPS)

S2 Fig LacZ expression section analysis of *Hbp1*^*lacZ_1*^. LacZ expression was detected in sections of 14.5 dpc ***Hbp1***^***lacZ_***^***1*** testes, although resolution of cell type was not possible. Scale bar = 50μm; Meso = mesonephros.
(EPS)

S3 Fig LacZ expression analysis of *Hbp1*^*lacZ_2*^. LacZ expression, driven by the 2 kb *Hbp1* proximal promoter, was detected at embryonic stages 12.5 **(A)** and 14.5 dpc in wildtype (Wt) and transgenic (Tg) embryos **(B)**. Promoter activity was also detected in 14.5 dpc somatic tissues including the forebrain **(C)**, hair follicles and eye **(D)**, limb **(E)** and neural tube **(F)**. Expression was not detected in either testes or ovaries at 14.5 dpc **(G,H)**.
(TIFF)

S4 Fig Cellular localisation of HBP-genetrap variant. Myc-tagged HBP1-genetrap variant was transfected into HEK293 cells and immunostained for MYC expression which revealed cytoplasmic localisation of the tagged protein. Cells were counterstained with DAPI to identify the nuclei and were visualised using confocal microscopy. Scale Bar = 10μm.
(EPS)

S5 Fig Histological analysis of teratoma harvested from an *HBP1*-genetrap chimera at 6 months. Externally an enlarged growth was evident (**A**, arrow). Internally, the growth was highly vascularised and had grown into the wall of the abdomen (**B**, arrow). H&E staining of sections revealed a teratoma with no normal spermatogenic tubules present **(C)** but multiple differentiated somatic cell types **(D,E)**. Sale bar = 200μm.
(TIFF)

S6 Fig Immunohistochemical analysis of *Hbp1*^*+/+*^ and *Hbp1*^*-/-*^ embryonic gonads. H&E staining of XY *Hbp1*^*++-*^ and *Hbp1*^*-/-*^ gonads revealed comparable morphology of somatic and germ cells at timepoints 12.5, 14.5 and 16.5 dpc. Scale bar = 50μm.
(TIFF)

S7 Fig Immunohistochemical analysis of somatic markers in *Hbp1*^*+/+*^ and *Hbp1*^*-/-*^ embryonic gonads. Investigation of Sertoli cell marker AMH and Leydig marker 3ßHSD revealed visually similar fluorescence intensity and localisation in *Hbp1*^*+/+*^ and *Hbp1*^*-/-*^ 14.5 dpc gonads. E-CAD (E-cadherin) marks germ cells. Apparent differences in total cord number between genotypes are due to the artefact of section position and gonad orientation. Scale bar = 50μm.
(EPS)

S1 Table Primer names, sequences and product sizes (bp). (DOCX)
