# Supplementary figures and images for: Mice Lacking Hbp1 Function Are Viable and Fertile
Source: PLoS One. 2017 Jan 20;12(1):e0170576. doi: 10.1371/journal.pone.0170576 (PMC5249219; doi:10.1371/journal.pone.0170576)

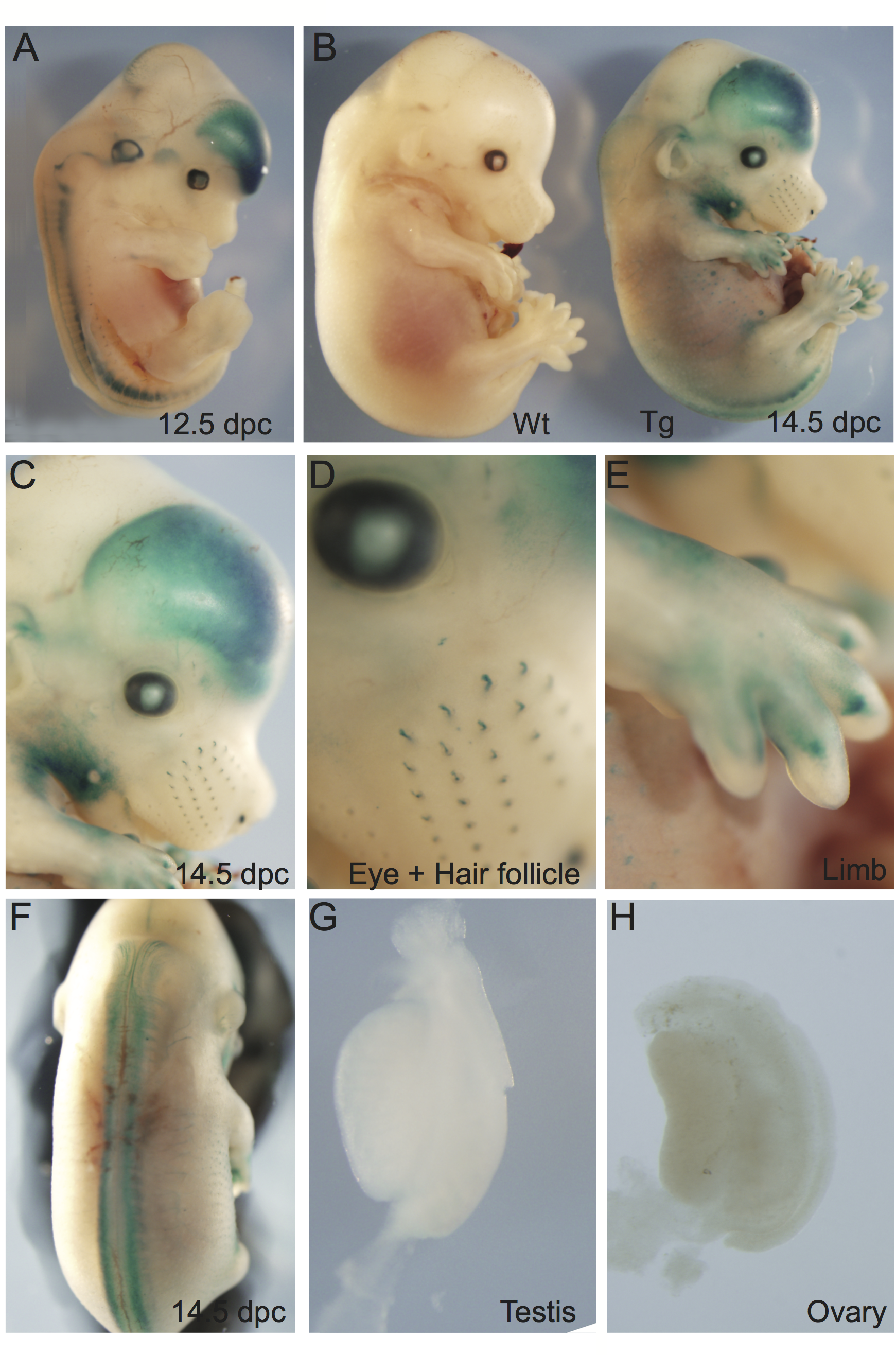

Supplement: S3 Fig — LacZ expression, driven by the 2 kb Hbp1 proximal promoter, was detected at embryonic stages 12.5 (A) and 14.5 dpc in wildtype (Wt) and transgenic (Tg) embryos (B). Promoter activity was also detected in 14.5 dpc somatic tissues including the forebrain (C), hair follicles and eye (D), limb (E) and neural tube (F). Expression was not detected in either testes or ovaries at 14.5 dpc (G,H). (TIFF) [file pone.0170576.s003.tiff]

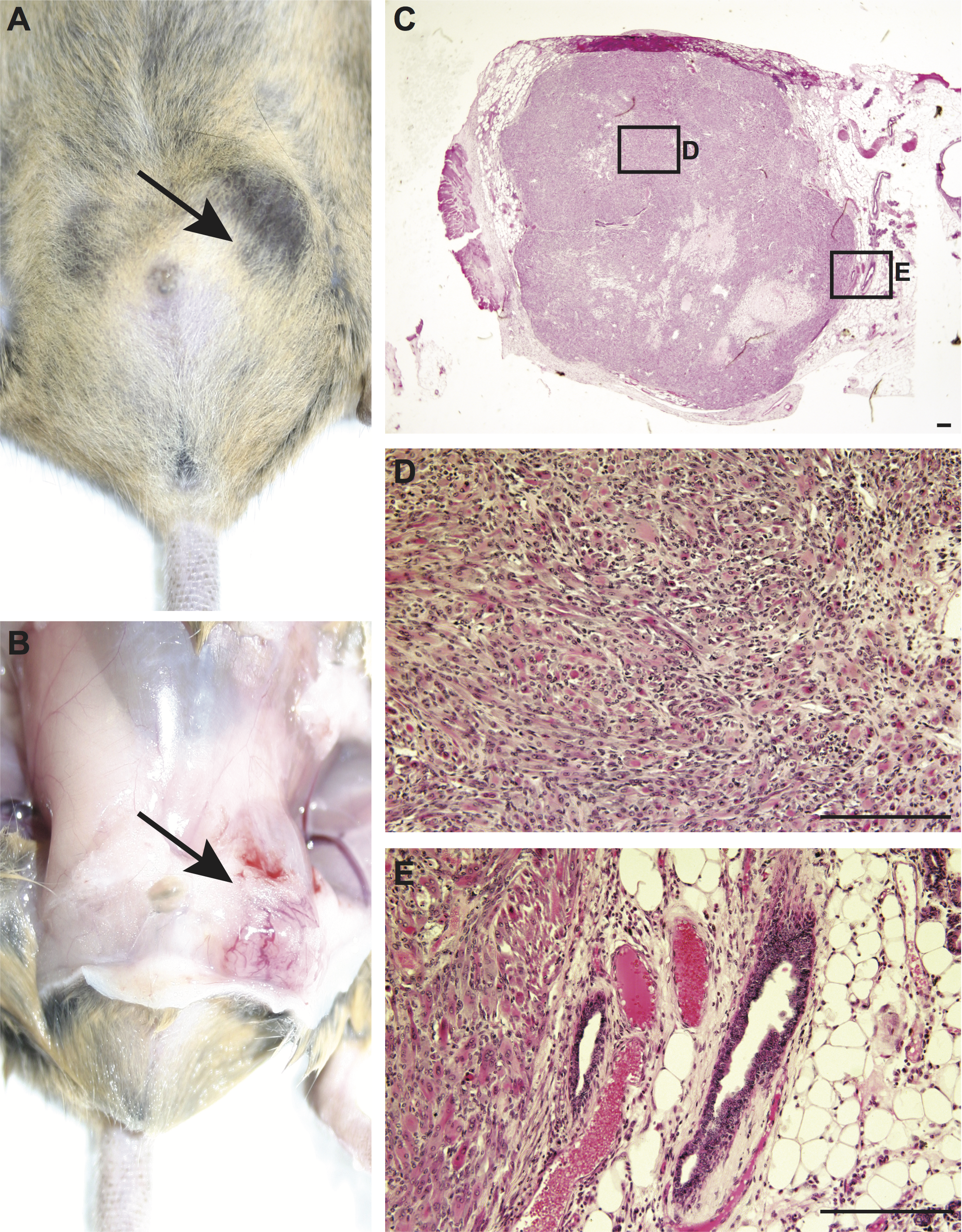

Supplement: S5 Fig — Externally an enlarged growth was evident (A, arrow). Internally, the growth was highly vascularised and had grown into the wall of the abdomen (B, arrow). H&E staining of sections revealed a teratoma with no normal spermatogenic tubules present (C) but multiple differentiated somatic cell types (D,E). Sale bar = 200μm. (TIFF) [file pone.0170576.s005.tiff]

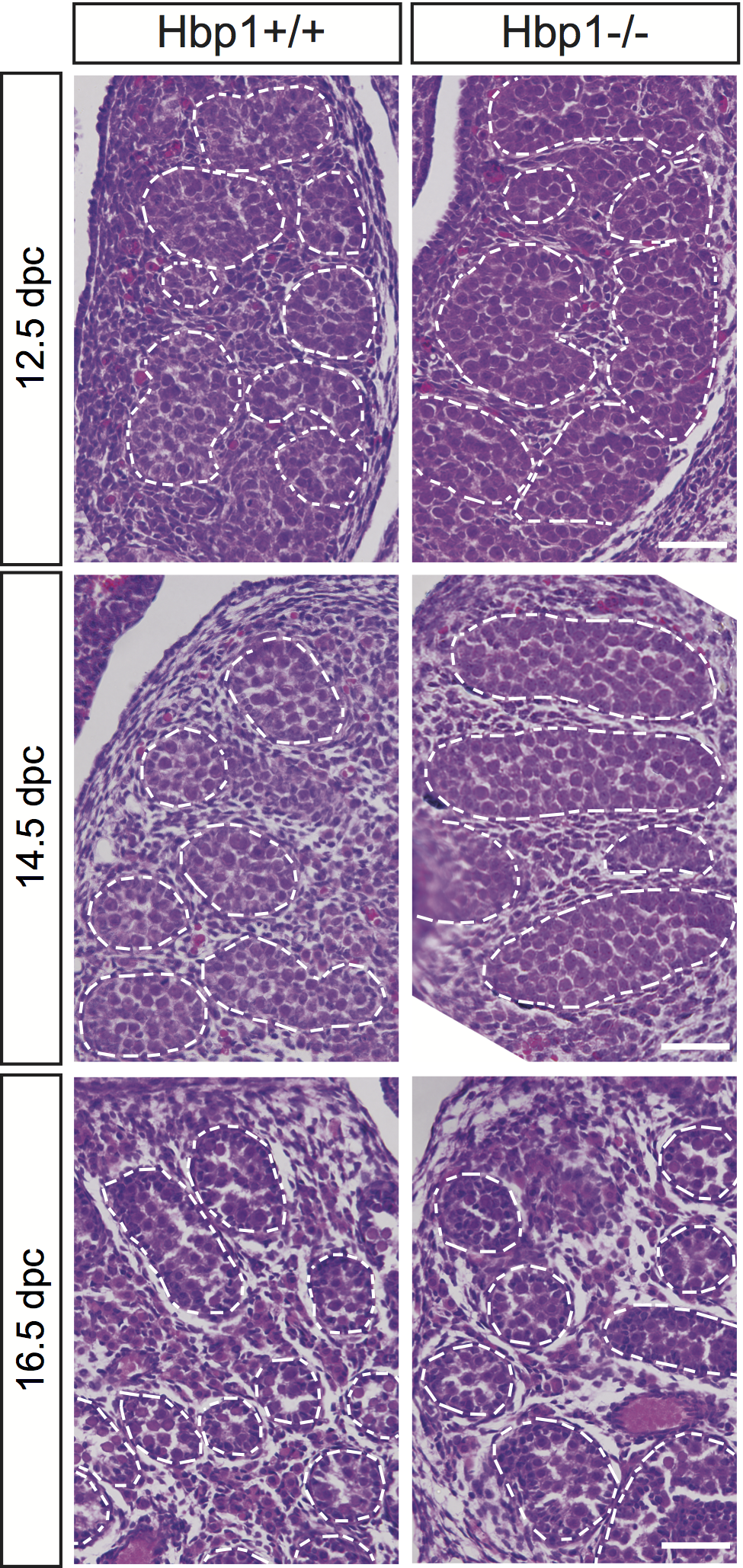

Supplement: S6 Fig — H&E staining of XY Hbp1++- and Hbp1-/- gonads revealed comparable morphology of somatic and germ cells at timepoints 12.5, 14.5 and 16.5 dpc. Scale bar = 50μm. (TIFF) [file pone.0170576.s006.tiff]
