# Supplementary material for: Mice Lacking Hbp1 Function Are Viable and Fertile
Source: PLoS One. 2017 Jan 20;12(1):e0170576. doi: 10.1371/journal.pone.0170576 (PMC5249219; doi:10.1371/journal.pone.0170576)
Supplement: S1 Table — (DOCX) [file pone.0170576.s008.docx]

**­­­S1 Table. Primer names, sequences and product sizes (bp)**

| **Primer** | **Primer sequence (5’-3’)** | **Product size (bp)** |
| --- | --- | --- |
| Fl-Hbp1-F | GACCATAAAATGCTGGGAGCAT | 903 |
| Fl-Hbp1-R | CAAGAATATAGTGTGCAAAATTGA |  |
| ∆Hbp1-F | CTTAAGAGTAGCTGTCTCATTT | 573 |
| ∆Hbp1-R | CTTGTGAAGTCCAAC ACATCAAACAA |  |
| FlqPCR-F | TTCCATGAATAGCCACCACA | 115 |
| FlqPCR-R | GACAATGACACCGACACCAT |  |
| ∆qPCR-F | AATGCCTTCATGCTTTTTGC | 115 |
| ∆qPCR-R | TGCCCTGACAAGTCAAGAGT |  |
| All-Hbp1-MYC-F | TCCAGCACAGTGGTGGTGTGGGAAGTGAAGACAAAT |  |
| »FlHbp1-MYC-R | GGCTCGAGTTAATGCTGTTGAGAGCCTGAGTT | 1550 |
| »∆Hbp1-MYC-R | GGCTCGAGTTACCTGTTATCTTTCCCTGGATA | 1394 |
| »Hbp1-genetrap-MYC-R | GGCTCGAGTTAGGCACTCACAGTCCCTGAGCT | 1289 |
| Hbp1P2kb-F | AAGGTACCCCCACTACGGCTTTTGGTAA | 2122 |
| Hbp1P2kb-F | AACCATGGTCGTCCACCTTTCTCGTCTT |  |
| LacZ-F | CTTAATCGCCTTGCAGCACA | 180 |
| LacZ-R | CAGTATCGGCCTCAGGAAGA |  |
| Oct3/4-F | TGCGGAGGGATGGCATACTG | 140 |
| Oct3/4-F | GCACAGGGCTCAGAGGAGGTTC |  |
| Ccnd1-F | GAAAATCGTGGCCACCTG | 68 |
| Ccnd1-R | GCGGGAAGACCTCCTCTT |  |
| Ccnd2-F | GCTGTGCATTTACACCGACA | 64 |
| Ccnd2-R | CACTACCAGTTCCCACTCCAG |  |
| Ccnd3-F | CAAAGCCCAAGCAAAGAAAG | 130 |
| Ccnd3-R | TGCTCGCTGCTCCTTCTTAC |  |
| p21-F | TCCACAGCGATATCCAGACA | 60 |
| p21-R | GGACATCACCAGGATTGGAC |  |
| p27-F | AGTGTCCAGGGATGAGGAAG | 73 |
| p27-R | TCTGTTCTGTTGGCCCTTTT |  |
| p57-F | CAGGACGAGAATCAAGAGCA | 118 |
| p57-R | GCTTGGCGAAGAAGTCGT |  |
| Ex5_Geo-F | TCGTCCACCTTTCTCGTCTT | 310 |
| Ex5_Geo-R | CAGTATCGGCCTCAGGAAGA |  |
| Mvh-F | CAGGAATGCCATCAAAGGAACAAC | 119 |
| Mvh-R | CCCAACAGCGACAAACAAGTAACTG |  |
| p63-F | TTCATCTGCTTTTCCCTCTCA | 61 |
| p63-R | GACCTGATGAAATATTGACTTCCA |  |
| Fgf9-F | CTATCCAGGGAACCAGGAAAGA | 119 |
| Fgf9-R | CTCGTTCATGCCGAGGTAGAG |  |
| Sox9-F | AGTACCCGCATCTGCACAAC | 145 |
| Sox9-R | TACTTGTAATCGGGGTGGTCT |  |
| Rb-F | GCCTCTCCAGCCTAACCATA | 63 |
| Rb-R | TTCTTTGGAGATCTTAGAGGAGAAA |  |
| p130-F | GGGAGACATGGATTTATCTGGT | 72 |
| p130-R | GCCAAGAGTGACCTGTGGA |  |
| p107-F | GCGGCAACTACAGCCTAGA | 81 |
| p107-R | TGCGGCAAGCAACATATAAA |  |
| p53-F | ACGCTTCTCCGAAGACTGG | 67 |
| p53-R | AGGGAGCTCGAGGCTGATA |  |
